# 

**Published:** 1914-02-14

**Authors:** 


### BEQUEST TO "BART.'S."

Mr. John Moore Walker, M.B., of Edgbaston, formerly medical officer to the Cheltenham Provident Dispensary, has left net personalty £45,372. The testator left £13,500 in personal legacies, and the residue of his property to St. Bartholomew's Hospital, which will apparently receive over £30,000.

### APPRECIATION OF A SCHOOL INSPECTOR.

The Acton Education Committee have increased the salary of Dr. Elsie Chubb (assistant school medical inspector) from £250 to £300, and have decided that her future increments be £50 to a maximum of £400. Dr. Chubb has only been in the service of the Acton Education Committee some nine months, but has already given great satisfaction.

RATES OF SUBSCRIPTION: Twelvemonths, 6/6; Six months, 4/-; Three months, 2/-, post free to any address in the United Kingdom. Abroad, Twelvemonths, 8/8; Six months, 5/-; Three months, 2/6, post free.—Address The Subscription Dept., "THE HOSPITAL," The Hospital Building, 28/29 Southampton Street, Strand, London, W.C.

